# Towards Validating the Effectiveness of Obstructive Sleep Apnea Classification from Electronic Health Records Using Machine Learning

**DOI:** 10.3390/healthcare9111450

**Published:** 2021-10-27

**Authors:** Jayroop Ramesh, Niha Keeran, Assim Sagahyroon, Fadi Aloul

**Affiliations:** Department of Computer Science and Engineering, American University of Sharjah, Sharjah 26666, United Arab Emirates; g00057302@aus.edu (N.K.); asagahyroon@aus.edu (A.S.); faloul@aus.edu (F.A.)

**Keywords:** electronic health records, machine learning, obstructive, polysomnography, prediction, sleep apnea

## Abstract

Obstructive sleep apnea (OSA) is a common, chronic, sleep-related breathing disorder characterized by partial or complete airway obstruction in sleep. The gold standard diagnosis method is polysomnography, which estimates disease severity through the Apnea-Hypopnea Index (AHI). However, this is expensive and not widely accessible to the public. For effective screening, this work implements machine learning algorithms for classification of OSA. The model is trained with routinely acquired clinical data of 1479 records from the Wisconsin Sleep Cohort dataset. Extracted features from the electronic health records include patient demographics, laboratory blood reports, physical measurements, habitual sleep history, comorbidities, and general health questionnaire scores. For distinguishing between OSA and non-OSA patients, feature selection methods reveal the primary important predictors as waist-to-height ratio, waist circumference, neck circumference, body-mass index, lipid accumulation product, excessive daytime sleepiness, daily snoring frequency and snoring volume. Optimal hyperparameters were selected using a hybrid tuning method consisting of Bayesian Optimization and Genetic Algorithms through a five-fold cross-validation strategy. Support vector machines achieved the highest evaluation scores with accuracy: 68.06%, sensitivity: 88.76%, specificity: 40.74%, F1-score: 75.96%, PPV: 66.36% and NPV: 73.33%. We conclude that routine clinical data can be useful in prioritization of patient referral for further sleep studies.

## 1. Introduction

Sleep research is of pertinence due to its fundamental role in ensuring health and wellbeing, and as cited by the American Psychiatrist Allan Hobson “Sleep is of the brain, by the brain and for the brain” [1]. Sleep disorders are impairments of sleep architecture (consisting of sleep stages) and disrupts psycho-physical health leading to the development of a host of diseases. More than a billion adults globally between the ages of 30 to 69 years suffer from obstructive sleep apnea (OSA), the most common type of sleep-disordered breathing. 936 million of them suffer mild to moderate symptoms and 425 million suffer from moderate to severe symptoms. The highest concentration of these individuals can be found in China, followed by India, Brazil, United States of America, Pakistan, Russia, Nigeria, Germany, France and Japan [2].

OSA causes temporary lapses in breath when the upper airway at the back of the throat becomes partially or completely blocked during sleep. This can lead to fragmented sleep since the individuals need to be conscious enough to wake up and reopen their airway to resume breathing and sleep and this poor quality of sleep results in sleepiness, fatigue and considerable physiological and psychological distress. Some of the common symptoms that can help identify the disorder is disrupted breathing, excessive daytime sleepiness (EDS), morning headaches, irritability, limited attention span, snoring and dry mouth [3]. Untreated OSA has been associated with many health conditions such as obesity, cardiovascular and metabolic disorders, in addition to reduced quality of life and depression [4].

To diagnose OSA, polysomnography (PSG) conducted in a sleep laboratory is usually considered as the gold reference standard. PSG monitors and records several body functions during sleep. If there are more than 15 obstructive respiratory events per hour of sleep, then no other symptoms are needed. The PSG test defines an apnea-hypopnea index (AHI) based on the criteria above. Severity grading varies, but typically mild OSA is defined by an AHI of 5 ≤ 15, moderate OSA by AHI between 16 ≤ 29, and severe by AHI ≥ 30. This method has several limitations: (i) it is expensive and time-consuming and requires medical supervision and in addition to being confined within a hospital or clinical setting, (ii) the sleep environment will be altered and does not represent the natural sleep context of the individual, and (iii) it cannot be implemented over a long time, being limited to a span of few days. There are other tests such as the multiple sleep latency test (MSLT), maintenance of wakefulness test (MWT), CPAP titration test, all of which are conducted in a controlled environment, typically following the PSG. Home sleep tests are a limited PSG which can be taken at home allowing it to be in the patient’s natural environment but it cannot determine sleep stages or other parameters which puts them at a major disadvantage. Self-assessment methods like sleep questionnaires and sleep diaries are an alternative inexpensive method which preserves the normal sleep environment but are highly subjective. Furthermore, sleep questionnaires are subject to bias due to patient reluctance in disclosing sensitive private information, or as a consequence of diminished awareness about the implications of potential sleep disorders. Sleep diaries contains more pertinent information as it is filled over a longer period of time, but has the same underlying issues as sleep questionnaires [5].

Accounting for these considerations, it is integral to develop easy-to-use and cheap accurate screening tools that can easily monitor disturbances in the population at a relatively low cost. In today’s increasingly digital world, there is a large amount of health data generated by different sources such as real-time physiological data from connected wearables, electronic health records (EHR), insurance claims and social media posts. Artificial intelligence, more specifically machine learning (ML) is emerging as a powerful tool in healthcare to mine available patient data and build powerful diagnostic frameworks [6]. This paradigm is gaining momentum in the area of OSA classification with two of the aforementioned sources: physiological data and EHR.

Physiological data can be derived from electroencephalogram [7], electrocardiogram or photoplethysmogram readings acquired either during PSG or through consumer-grade wearable devices [8]. In general, the former type of data collected in sleep labs with a ground truth respiratory signal achieve noticeably better performance with any ML algorithms. While actigraphy studies are attractive owing to its applicability in community based populations, it is inherently challenging to achieve comparable OSA screening performances as those from sleep lab studies. This is a consequence of occurrences such as noise, motion artifacts or other disturbances (such as battery depletion, missing data, loose skin contact, etc.). Researchers have also developed smartphone sensor based application for sleep apnea monitoring [9] and presented contact-less sleep disorder detection using sonar techniques [10]. The physiological monitoring modalities have the common issue of requiring additional obtrusive monitoring apparatus or expert supervision, which brings to the forefront the alternative approach of using routinely acquired electronic health records to perform screening. In can be surmised that sleep physiological data such as pulse oximetry and sleep stage duration have considerable predictive ability, but are not readily available, as the expensive, time consuming and labor intensive nature of PSG limits regular monitoring and diagnosis [11,12]. Moreover, the variability in performance of such solutions over an extended period of time within a community based setting conveys a relatively low level of overall reliability.

The use of digital health records and machine learning techniques trained on Big Data publicly available can allow for the transfer the knowledge representation to generalized cases. These tests would be more accurate in identifying patients with a higher pretest probability of OSA and can rule out OSA in low-risk patients, due to the high volume, veracity, velocity, variety and value provided by the datasets [4]. There are multiple successful studies leveraging EHRs to implement effective disease prediction models in literature [13]. A study conducted using EHRs from over 1 million outpatient visits from over 500,000 patients at a major academic medical referral center in China, was used to create an AI-based diagnostic system for detection of pediatric diseases with an accuracy in the ranges of 90–95% for multiple disease categories [14]. Although traditionally predictive modelling techniques require custom datasets, with specific variables limit the scope of the applicability, especially with large feature variables, recent developments in artificial intelligence address these challenges [15]. Predictive modeling with electronic health records using the “transfer learning” approach has shown to accurately predict medical events from multiple clinics without being site specific [16]. Moreover, with the creation of flexible standardized clinical data representation formats like FHIR (Fast HealthCare Interoperability Resources), any developed models can be integrated into clinical systems [17]. One of the primary advantages of such models would be the ability to contribute to a wider population health paradigm using the routine biomarkers and patient profiles in hospitals to screen and preemptively identify at risk individuals for care. These screening methods reduce the need for patients to undergo either obtrusive tests such as PSG to even identify sleep disorders, or remote patient monitoring systems using wearables, although these approaches do have their value in screening within consumer lifestyle management applications. There is a significant cost reduction to both the clinics and patients in the deployment of clinical screening algorithms, as they would not be as expensive as PSG, and allows for consideration of patients who do not have wearable devices as well. Most literature in this intersecting area of patient health records, Big Data and deep learning focus on prediction of mortality, cardiovascular risks, diabetes and pulmonary conditions. A systematic review of recent developments in deep learning methods and their clinical outcomes with the utilization electronic health records can be observed in [18]. Their study reiterates that general conditions such as suicide risk, future disease predictions, readmission probability prediction, heart failure prediction and hospital stay duration estimation are the actively researched areas.

The experiments in [19] saw the deployment of a learning algorithm to distinguish cases of diagnosed OSA and non-cases using EHR ICD-codes across six health systems in the United States. A cohort study of adults in Canada was conducted as follows in [20], where an algorithm trained on administrative data and ICD-codes found a high degree of specificity in identifying patients with OSA. A super sparse linear integer model was developed in [21], by training the model on self-reported symptoms, self-reported medical information, demographics and comorbidities data to screen for OSA cases with considerably success. Another study [22] focused on developing a support vector machine-based prediction model using 2 to 6 features collected at clinical visits to identify patients with AHI index at 3 cut offs. The model was fivefold-cross validated and had balanced performance measures in the 70% range. It outperformed the Berlin Questionnaire, NoSAS score and Supersparse Linear Integer model for the age category for men below 65 years of age. The primary limitations between the clinical data trained models are due to oversampling of the target class (i.e., more sleep apnea cases than control group), lack of generalizability (due to limited data features), and relatively high false alarms for OSA [23]. In clinics where PSG is not possible, or there is no sleep data available, medical staff still screen using self-reported questionnaires during patient visits [24]. There is room for improvement, especially considering boosting algorithms as their ability to uncover non-linear patterns are unparalleled, even given large number of features, and make this process much easier [25].

This work presents and attempts to answer this question: “Is it possible to develop machine learning models from EHR that are as effective as those developed using sleep physiological parameters for preemptive OSA detection?”. There exist no comparative studies between both approaches which empirically validates the quality of using routinely available clinical data to screen for OSA patients. The proposed work implements ensemble and traditional machine learning models to screen for OSA patients using routinely collected clinical information from the Wisconsin Sleep Cohort (WSC) dataset [26]. WSC includes overnight physiological measurements, and laboratory blood tests conducted in the following morning in a fasting state. In addition to the standard features used for OSA screening in literature, we consider an expanded range of questionnaire data, lipid profile, glucose, blood pressure, creatinine, uric acid, and clinical surrogate markers. In total, 56 continuous and categorical covariates are initially selected, the the feature dimension narrowed systematically based on multiple feature selection methods according to their relative impacts on the models’ performance. Furthermore, the performance of all the implemented ML models are evaluated and compared in both the EHR and the sleep physiology experiments.

The contributions of this work are as follows:

- Implementation and evaluation of ensemble and traditional machine learning with an expanded feature set of routinely available clinical data available through EHRs.
- Comparison and subsequent validation of machine learning models trained on EHR data against physiological sleep parameters for screening of OSA in the same population.

This paper is organized as follows: Section 2 details the methodology, Section 3 presents the results, Section 4 discusses the findings, and Section 5 concludes the work with directions for future research.

## 2. Materials and Methods

**Figure 1:**
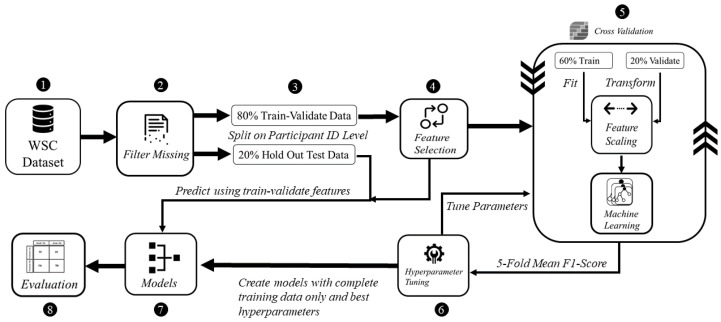
High level view of the proposed methodology.

As shown in Figure 1, the proposed methodology composes of the following five steps: (i) preprocessing, (ii) feature selection, (iii) model development, (iv) hyperparameter tuning and (v) evaluation. This process is conducted for the EHR as well as for the physiological parameters acquired from the same population in the WSC dataset.

OSA is a multi-factorial condition, as it can manifest alongside patients with other conditions such as metabolic, cardiovascular, and mental health disorders. Blood biomarkers can therefore be indicative of the condition or a closely associated co-morbidity, such as heart disease and metabolic dysregulation. These biomarkers include fasting plasma glucose, triglycerides, and uric acid [27]. The presence of one or the other comorbidities does not always necessarily indicate OSA, however in recent literature clinical surrogate markers reflective of particular conditions have shown considerable association with suspected OSA. Clinical surrogate markers exhibit more sensitive responses to minor changes in patient pathophysiology, and are generally more cost-effective to measure than complete laboratory analysis [28]. Thus, we derive 4 markers, Triglyceride glucose (TyG) index, Lipid Accumulation Product (LAP), Visceral Adipose Index (VAI) and the Waist-Height Ratio (WHrt), and observe their value in discriminating between OSA and non-OSA patients [29]. Ref. [30] reports LAP, VAI and TyG were reliable surrogate markers for identifying metabolic syndrome in middle-aged and elderly Chinese population. TyG was independently associated with increased OSA risk, as it is a reliable marker of insulin resistance, comprising of glucose intolerance, dyslipidemia, and hypertension [31]. This relationship is observed as insulin resistance increases due to the intermittent periods of asphyxia, hypoxia and sleep depivation caused due to OSA [32].

The Wisconsin Sleep Cohort (WSC) from University of Wisconsin-Madison is a study of 1500 participants having the causes, consequences and natural history of sleep disorders [26]. Fifty-six total features are extracted and categorized into demographics, anthropometry, blood tests, derived clinical markers, general health questionnaires, self-reported history, polysomnography derived parameters, as presented in Table A1, Table A2, Table A3, Table A4, Table A5, Table A6, Table A7 and Table A8 respectively within Appendix A. The dataset contains 2570 records of the 1500 participants assessed at four-year intervals, where each participant can have up to five records in the study. The total number of participants/patients is denoted by $n_{p}$, and the total number of health records is denoted by $n_{r}$. The demographics included age, sex, race, alcohol and smoking habits. The anthropometric features included patient height, weight, BMI, waist circumference, and neck circumference. The laboratory blood test results were obtained the morning following the overnight sleep study in a fasting state. The profiles are of fasting plasma glucose, HDL-C, LDL-C, total cholesterol, creatinine, uric acid, systolic and diastolic blood pressure. The self-reported history consisted of general health status, existing medical conditions and sleep symptoms, which were acquired through self-administered questionnaires. Finally, polysomnography derived parameters included objective information about sleep stages, sleep duration, AHI events, and oxygen saturation levels. To compare model discriminability when trained with clinical data features and PSG parameters, they are used exclusively to implement independent models.

An eighteen channel PSG system (Grass instruments model 78; Quincy, MA, USA) was used to record sleep state with electroencephalography, electrooculography, and electromyography [33]. Breathing, nasal and oral airflow, and oxyhemoglobin saturation were assessed respectively using respiratory inductance plethysmography (Respitrace; Ambulatory Monitoring, Ardsley, NY), thermocouples (ProTec, Hendersonville, TN and Validyne Engineering Corp pressure transducer, Northridge, CA) and pulse oximetry (Ohmeda Biox 3740; Englewood, CO, USA) [33]. Every 30 s of the PSG recordings were scored in terms of sleep stage and apnea and hypopnea events by trained technicians according to conventional standards [34,35]. Cessation of airflow for ≥10 s and discernible reduction in breathing expressed as a sum of chest and abdominal excursions with a oxyhemoglobin saturation decrease of ≥4% defined apnea and hypopnea events respectively [33].

The dataset was examined for missing values for deletion or imputation. Little’s MCAR (Missing Completely at Random Test) confirmed the null hypothesis (p>0.05) that the pattern of missing values did not have any significant relationship with the rest of the data [36]. As such, imputation would not be an effective approach, due to the large number of missing values in the records relative to the total size of the dataset itself. Thus, listwise deletion was employed to remove entire records where the clinical features of interest values were missing, or had a numeric value of 0 where domain knowledge states it is not possible (e.g., fasting plasma glucose, triglycerides). Continuous variables and categorical variables were handled separately, due to their differing mathematical characteristics. Continuous variables were scaled using the standardization technique to distribute the values around a mean with unit standard deviation. Categorical variables were converted into one-hot encoded vectors equal to the number of unique categories for each column using dummy variables.

The data records were split on a participant level into a training-validation set consisting of distinct patients ($n_{p}=752$) and a hold-out testing set of ($n_{p}=188$) patients. The cleaned dataset had ($n_{r}=1479$) records, where ($n_{r}=853$) records exhibited OSA and ($n_{r}=626$) did not have OSA. This was done as a single patient can have multiple records in the dataset, and records repeating across the both training set and testing set will introduce data leakage.For the development of both the EHR and PSG data based models, the same training-validation and hold-out sets are used. All subsequent analysis that are part of steps (i)–(iv) in the methodology is conducted using the training-validation split, and step (v) is applicable for the hold-out testing set.

The populations were split at the threshold of AHI =5 for the total of 56 features. In all following analysis, *p*-values < 0.05 are the cut-off for statistical significance. We applied the Shapiro-Wilk test of normality [37] to the populations, and note deviation from Gaussian distribution. Hence, we apply the Mann Whitney U-Test [38], which is distribution agnostic, to the continuous variables. Only self-reported sleep latency, LDL-C, total cholesterol, creatinine, Horne Ostberg score, State-Trait anxiety scores, non-REM sleep duration, and percentage of sleep stage 3&4 had *p*-values > 0.05. The average age is above 50 for both populations, and it is more probable that some of the patients may be facing onset of age-related diseases and increasing risk of OSA [39]. However, despite the aging, the overall population appears to be healthy, without much severity in any present comorbidities.

For categorical variables, we apply Chi Square with Bonferroni-Adjusted-*p*-value, as post-hoc testing can reduce false positives when multiple category levels are involved. No Yates correction was employed, to yield conservatives in the obtained *p*-values [40]. The demographic is heavily skewed towards the Caucasian ethnicity. Other perceived differences are in distribution of sexes (more men), occurrences of previous heart attacks, hypertension issues, angina, coronary, diabetes, arthritis, congestive heart failure, existing apnea and excessive daytime sleepiness along with snore volume being relatively higher among the OSA group. In terms of lifestyle, alcohol consumption and smoking is fairly similar between the two populations.

Feature selection was conducted using only the training-validation set. To mitigate possible selection bias and reduce redundancy, consistently highly ranking common features across all feature selection methods are chosen. We run two variations of this approach to ascertain the relative importance of all features. The intersection of the top two and top twenty features from each method is taken in the two cases respectively. The lower and upper bounds for the top features experiment is decided based on the distribution of the feature importance scores. To be more specific, many features have approximately the same impact on the AHI values, and we demarcate the two points where the differences between subsequent scores are the highest.

In the feature selection process for the clinical data, biological plausibility and their effective values during correlation with OSA were considered as well [41]. Automated step-wise procedures were avoided in favor of manual feature selection to ensure that the predictions made by the model can remain interpretable by medical professionals, if needed.

**Figure 2:**
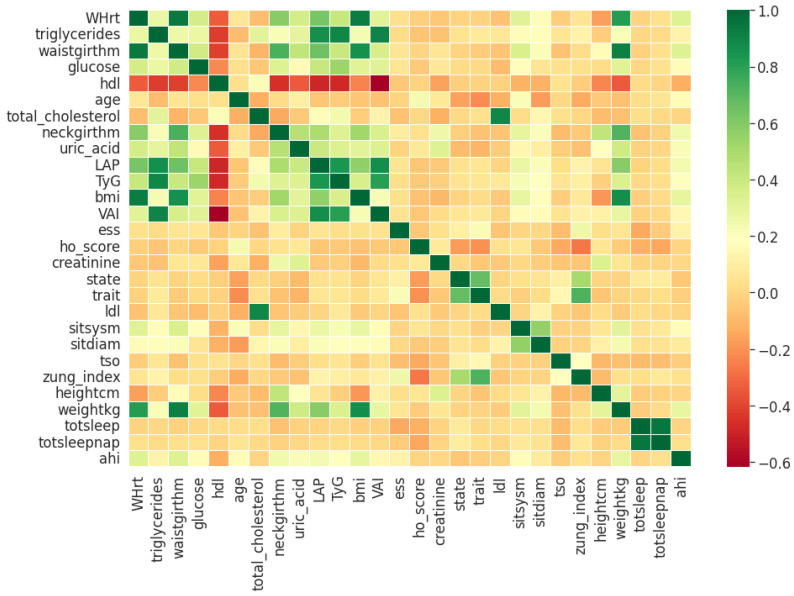
Clinical features ordered as per Pearson’s Correlation Coefficient.

**Figure 3:**
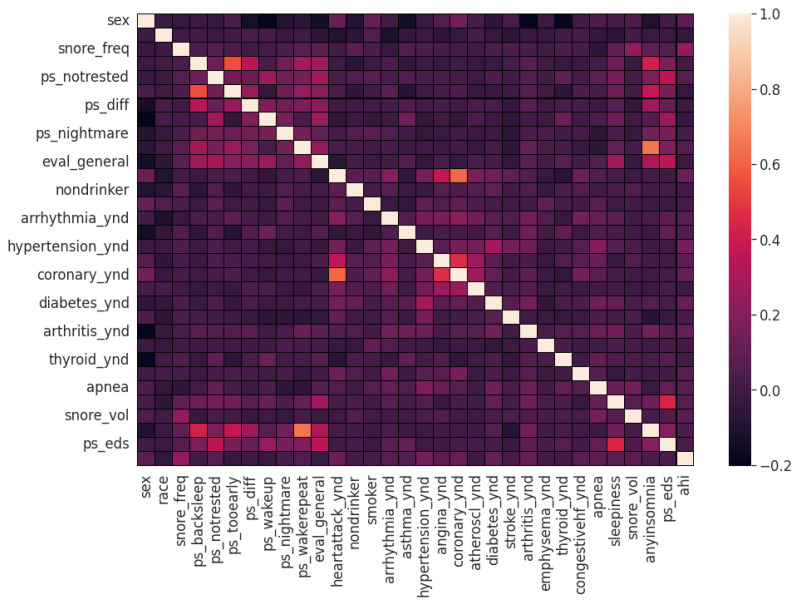
Clinical features ordered as per Kendall’s Tau.

**Figure 4:**
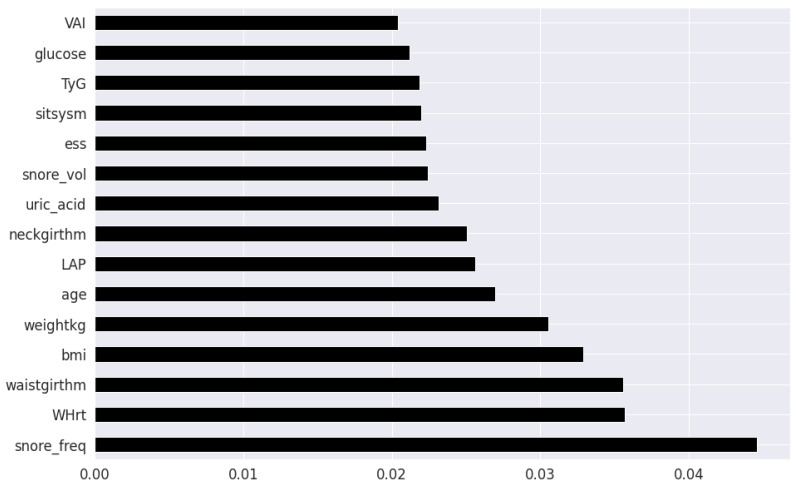
Clinical features ordered as per Extremely Randomized Trees.

**Figure 5:**
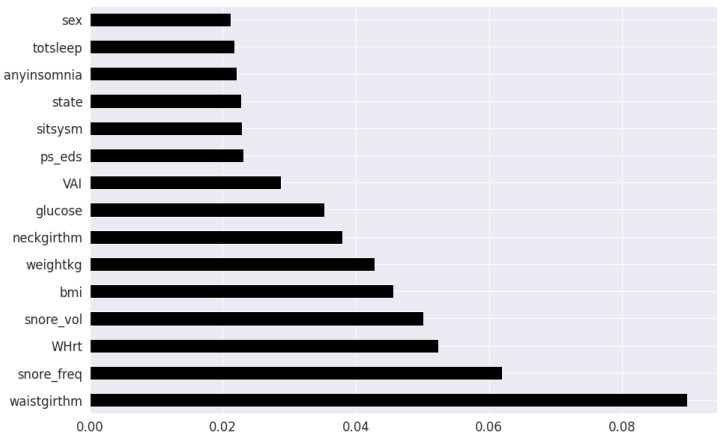
Clinical features ordered as per Mutual Information.

Pearson’s correlation coefficient estimates coefficients between the output class and each of the predictor features signifying the strength and nature of the relationship between the two [42]. The coefficient is distributed between −1 and +1, where the former is total negative correlation, and the latter is total positive correlation. 0 indicates no linear correlation between the variables. We select the continuous features with positive and negative correlation as per this method to capture linear relationships, as shown in Figure 2. The coefficient estimation does not assume normality, but does assume finite variance and finite covariance as per the central limit theorem. Kendall’s Tau correlation coefficient is a non-parametric test for measuring degree of association between the output class and predictor features applicable for categorical variables [42]. It is more robust to outliers and operates on the principles of comparing concordant and discordant pairs for ordinal variables. The most impactful categorical features are selected, as shown in Figure 3. Extremely Randomized Trees Classifier is a method where a number of randomized decision trees are fitted on subsets of the dataset [43]. Each decision tree results in a different model that has been trained with a different set of features. The relative importance of each feature on the classification performance of AHI is quantified as per the Gini index, as shown by Figure 4. We apply the Mutual Information technique to ensure that all strong associations, even non-linear between the continuous and categorical features with respect to the output class of OSA have been effectively captured [44]. Information gain measures the reduction in entropy of predictor features by partitioning a dataset according to the output classes. The entropy quantifies the probability distribution of observations in the dataset belonging to positive or negative class. Higher information gain suggests higher dependency between a feature and a specific output, while 0 suggests both are independent of each other. This method accepts continuous and categorical variables, and is able to capture both linear and non-linear relationships, as shown in Figure 5.

The final feature set in the top two-features per method consisted of a total of 8 features: waist circumference, neck circumference, daily snoring frequency, snoring volume, EDS, BMI, Whrt, and weight. The final features in the top twenty-feature per method consisted of the following 11 features in addition to the previous 8 features: fasting plasma glucose, LAP, uric acid, VAI, hypertension, heart attack comorbidity, TyG, triglycerides, systolic blood pressure and age.

**Figure 6:**
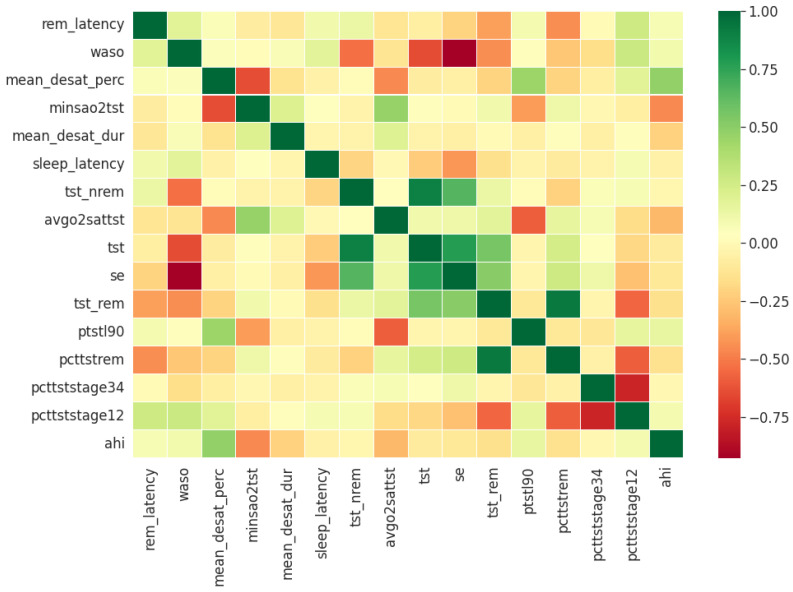
PSG features ordered as per Pearson’s Correlation Coefficient.

**Figure 7:**
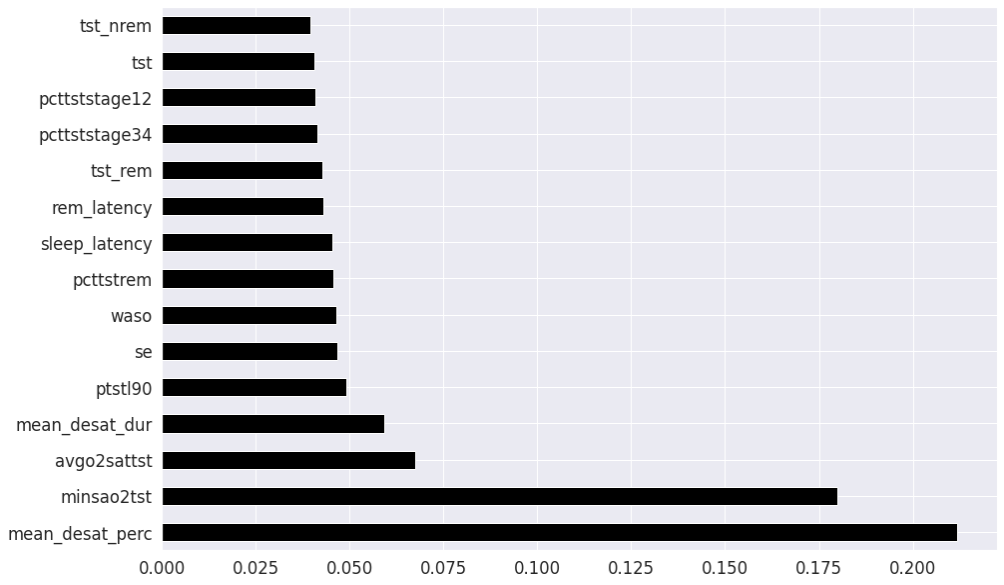
PSG features ordered as per Extremely Randomized Trees.

**Figure 8:**
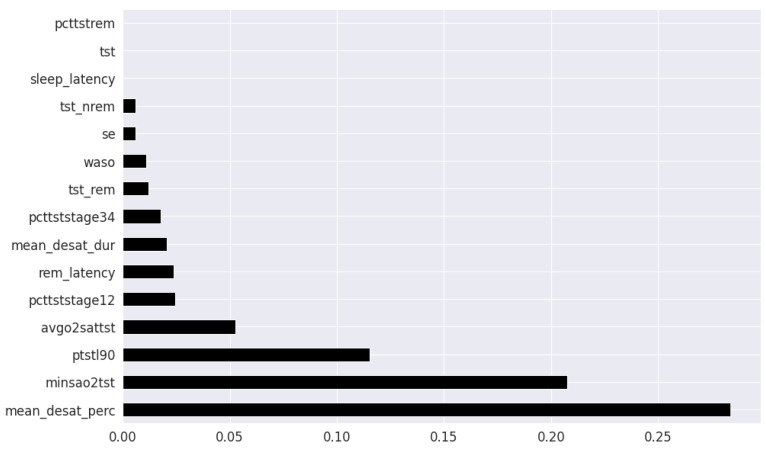
PSG features ordered as per Mutual Information.

In the feature selection process for the PSG parameters, all the variables were continuous. Thus, Kendall’s Tau was excluded, and the feature rankings from Pearson’s Correlation Coefficient, Extremely Randomized Trees Classifier, and Mutual Information are shown in Figure 6, Figure 7 and Figure 8 respectively. Unlike the clinical data features, where multiple features had relatively similar influences on the dependent AHI variable, the most important parameters from PSG are the mean desaturation percentage, and minimum level of oxygen saturation. This is expected as the apnea-hypopnea events are scored using the changes in breathing and airflow.

The final feature set in the top two-features per method derived from oximetry consisted of a total of two features: mean oxygen desaturation percentage, and minimum level of oxygen saturation. The final feature set in the top fifteen-features per method derived from oximetry c in the top fifteen features consisted of the following 4 features in addition to the previous 2 features: sleep duration with oxygen saturation percentage below 90%, REM sleep latency, average oxygen desaturation of apnea-hypopnea event and mean oxygen desaturation duration.

Ensemble methods include “bagging” (e.g., Random Forest algorithm) and “boosting” methods (e.g., Extreme Gradient Boosting technique). Ensemble machine learning methods such as gradient boosting iteratively combines a set of weak base classification models to construct a strong learner. Gradient boosting techniques are currently being employed to attain state-of-the-art results in clinical applications [45,46]. Gradient boosting techniques sequentially minimize the residual error of preceding learners. The variation in individual base learner configuration is expected to capture different relationships in the data distribution. Its integration into a unified prediction model is similar to the concept of collecting various expert opinions on an initial prognosis, aggregating and making a final decision.

Extreme gradient boosting (XGB) [47] utilizes the gradient boosting framework, with the algorithmic enhancements of regularization, sparsity awareness, weighted quantile sketch and internal cross-validation. Light gradient booting machine (LGBM) [48] is another variant, where the key difference is in its implementation of vertical decision tree growth and gradient-based One-Side Sampling strategy. LGBM grows tree in a leaf-wise manner, as opposed to level-wise, thereby is capable of reducing delta loss more drastically. CatBoost (CB) [49] is yet another variant of gradient boosting, with the refinement strategies of symmetric tree implementation, ordered target statistics and ordered boosting to minimize prediction shift with categorical variables.

The traditional machine learning models of k-Nearest Neighbours (kNN), Support Vector (SVM) Machines and Logistic Regression (LR) are used as baseline to benchmark the performance of the ensemble techniques [50]. KNN is non-parametric learning algorithm which distributes similar instances in the same proximity defined by the Euclidean distance, and classifies new unknown instances by majority vote of their *k* nearest instance neighbours. SVM is an algorithm that performs prediction by optimally separating the data instances of different classes in an *n* dimensional space using a hyperplane and its associated support vectors. LR is an extended case of the classic linear regression method, in which one or more independent input variables predicts the probability of occurrence of a binary output variable.

We applied a hybrid hyperparameter tuning approach by combining a Bayesian Optimization variant for global search, and a genetic algorithm for local search. The methods were Tree-structured Parzen estimator (TPE) [51] and Covariance matrix adaptation evolution strategy (CMA-ES) [52] respectively. TPE constructs a probability model of the specified objective function, and identifies the ideal hyperparameters, and CMA-ES iteratively samples candidate solutions using a derivative free approach. The parameters and instantiation values for both the algorithms are based on the work presented in [53]. The optimization criteria was the aggregate cross-validation F1-score of the training-validation set in order to achieve a balanced screening system.

## 3. Results

All analysis were conducted using Python 3.7.12 on a workstation operating a Linux OS with 24 GB RAM, Intel Quad-Core Xeon CPU (2.3GHz), and Tesla K80 GPU (12 GB VRAM). The Python libraries used are mentioned in the subsequent paragraph.

Data was processed with numpy 1.19.5 [54] and pandas 1.1.5 [55]. Statistical methods and correlation tests were performed using scipy 1.4.1 [56]. Gradient boosting models were constructed using the standard xgboost 0.90 [47], lightgbm 2.2.3 [48] and catboost 1.0.0 [49] libraries. Baseline machine learning models were constructed using scikit-learn 1.0.0 [57]. Visualizations were made using seaborn 0.11.2 [58] and matplotlib 3.2.2 [59]. Hyperparameter tuning was performed using the Optuna 2.10.0 library [53].

The following metrics are used to ascertain the performance quality of the gradient boosting models through a 5-fold cross-validation approach: accuracy (Acc), sensitivity (Sen), specificity (Sp), positive prediction value (PPV), negative prediction value (NPV), F1-Score, and Area Under Curve (AUC). Accuracy is the proportion of correct predictions across the total test dataset. Sensitivity is the proportion of OSA patients correctly identified as positive and specificity is the proportion of non-OSA patients correctly identified as negative. Positive prediction value is the probability of positive cases correctly being OSA patients, and negative prediction value is the probability of negative cases correctly being non-OSA patients. The F1-score measures the balance between positive predictive value (cause of type-1 errors) and sensitivity (cause of type-2 errors). Area Under Curve denotes the trade-off between sensitivity and specificity, with the cut-off value identified using the Youden index.

All reported metrics of the EHR trained and oximetry trained models are obtained through evaluation on the hold-out test data in Table 1, Table 2, Table 3, Table 4 and Table 5. The best hyperparameters used to generate the reported results in Table 1 and Table 4 are provided in Table A9 and Table A10 respectively.

It is observed that the oximetry related parameters exhibit a considerably better performance for detecting OSA across all metrics with its increased impact evident particularly on specificity, as evident by Table 3. These features are capable of finding patterns whilst remaining fairly stable in small amounts of data as well, which may required for data constrained environments. Since trained specialists perform annotation of an apnea or hypopnea event based on the nature of respiration and oxygen levels, it is expected that the respective physiological parameters reflecting this are much more effective. However, in non-monitored, community-based conditions where patient apnea events are classified by automated algorithms through portable medical devices, smartphones or smart watches, the efficacy of alternate parameters needs to be examined further. Despite these observations, we can surmise that the routinely collected clinical features of waist circumference, neck circumference, BMI, and weight along with the self-reported symptoms of EDS, snoring frequency and snoring volume and derived clinical surrogate markers of lipid accumulation product and Waist-Height ratio have utility in identification of OSA. Thereby, in comparison with overnight pulse oximetry, use of electronic health records is a viable alternative, albeit for early risk screening and prioritization of OSA patients.

## 4. Discussion

The primary motivation behind the application of ensemble gradient boosting algorithms in this work was an attempt to capturing higher dimensional interactions in the data, as a consequence of the multifactorial nature of OSA. The performance of the SVM, LR, and KNN baseline models are relatively similar to the performance of boosting (CatBoost, XGB and LGBM) and bagging (RF) algorithms with the top 8 features as presented in Table 1. Interestingly, the ensemble models do not fare significantly better than the traditional models in either the EHR or PSG case. For the 8 feature case, the sensitivity, F1-score and NPV of the SVM is the highest, while LGBM has higher specificity, PPV and AUC. CB has the second highest sensitivity and F1-score. For the 19-feature case, the XGB model performs the best across the metrics of accuracy, sensitivity, F1-score, PPV, and NPV while LGBM still retains the highest specificity. SVM has the second highest sensitivity but its performance across the other metrics is not as comparable. However, as the number of features increase, roughly a factor of two in this case, the overall performance begins to decrease as presented in Table 2. The F1-score, a robust metric of reliability is consistently higher for the ensemble techniques in the 19 feature case. It is possible that in the case of non-linear relationships, ensemble learning can learn more complex relations from relatively small amounts of data (∼1000 samples). The intention behind selecting the most important 8 EHR features then extending to 19 EHR features, is to observe whether an increase in the number of EHR features with association to OSA can improve the specificity of detection. We note that age, triglycerides, and the existing conditions of hypertension and previous heart attack exhibit the ability to predict OSA, but it does not increase the rate of detection among the population sample available for this work. Since the focus of this work is identifying the model giving rise to the highest sensitivity for screening with the most impactful features, even at the expense of specificity, the SVM is most applicable. When we compare the EHR performance metrics to the PSG case, the disparity is evident in favor of the latter. As the number of features are increased in the PSG case, all metrics across all models exhibit a modest increase in performance. In both the 2 feature and 6 feature experiment, the CB model emerges as the best method, followed by RF. It is possible that in the EHR case that multiple features are related with each other, and there is underlying redundancy, which does not contribute towards the knowledge representation learned by the models. In contrast, the addition of more PSG features might be providing extra information, which enables the models with an improved representational understanding of the relationship between these predictors and OSA severity.

One of our contributions are in the expansion of the initial feature dimensions to 56 EHR parameters, consisting of a combination of medical history, comorbidities, clinical measurements, laboratory blood tests and self-reported symptoms. Most existing works only consider for waist circumference, neck circumference, BMI and age as the feature set, which may not completely represent the populations at risk of OSA. Risk factors underlying the decision remain poorly understood, therefore adding multiple dimensions, can potentially reduce the unnecessary referrals and account for the typically missing screening of patients with sleep apnea and minimal snoring. We additionally evaluate the role of LDL-C, HDL-C, fasting plasma glucose, uric acid and derived clinical surrogate markers of Whrt, LAP, VAI and TyG in predicting OSA, within a machine learning context. With the incorporation of additional features, we attempted to rectify the high false positive rate by increasing model specificity through holistic consideration of a complete patient medical history. Gradient boosting methods were applied with the intentions of reducing bias, improving generalization ability and reducing overfitting. Regardless, these models exhibit only marginal superiority over traditional methods such as SVM.

Waist, neck circumference and EDS have been long established as vital indicators for OSA susceptibility, and results of feature selection methods are in agreement. It is important to note that abdominal obesity is not the same as peripheral obesity. Waist circumference depends on the fatty tissues in the peritoneum, and thus, the abdominal obesity, which is known to affect upper airway functioning, a consistent symptom of OSA [62].

Frequent snoring was detected during feature selection as yet another pertinent feature for OSA prediction, and is part of the minimal feature set for the trained models. Although experts in [63] advise caution in the interpretation of snoring symptoms for assessing sleep apnea, they state it can be reliable when used in conjunction with additional clinical and physical readings, which is the case in our presented work. While the features of insomnia and daytime sleepiness (quantified by ESS) were included in feature selection, they only showed a marginal association with OSA, as opposed to the stipulations of [64,65], respectively. This can be explained by the overall minimal OSA severity levels of the dataset population used in this work.

Patient laboratory blood tests and clinical surrogate markers were introduced as auxiliary biomarker features and its value in improving the model discernibility for classification of OSA was studied. In the case where 19 features were utilized for training, fasting plasma glucose, uric acid, and LAP (dependent on on waist circumference to triglycerides ratios) showed correlation with OSA in a similar fashion to traditionally expected indicators such as EDS and BMI. Additionally, the clinical markers of systolic blood pressure, VAI, and TyG are also present. These biomarkers are associated with OSA, and is in concordance with prior literature. Although the models were not able to utilize all biomarkers relevant to OSA with equal effectiveness, the possible reasons for the findings and variations in this work are worth mentioning.

Fasting plasma glucose is arguably the strongest blood biomarker feature, ranking consistently highly behind the physical measurements and snoring features across all the feature selection methods. This is expected given its relation with sleep quality and the effect of fragmented sleep on metabolic dysregulation which causes elevated glucose levels in the body, as reported in [66]. For some patients, the presence of insulin resistance/glucose irregularity, overlaps with the OSA symptoms of upper airway narrowing and decrease reduced dilator muscle contraction. Interestingly, glucose irregularity in a sleep disordered population of males has been shown in [67] to be independent of obesity and diabetes, indicating a strong correlation with OSA severity. From the findings of [68], OSA was independently associated with decreased insulin sensitivity in a female population as well.

In this work, uric acid emerged as a viable secondary predictor for OSA. This is likely due to hyperuricemia, which is an excess of uric acid levels, has been reported to be significantly associated with OSA as well as obesity and overnight oxygen desaturation severity.

As hypothesized, it appears the Whrt and VAI and LAP indices prove to be useful indicated as well. This is expected since fat distribution, visceral fat, and body composition increases the risk of anatomical irregularities common among OSA patients, and this is stated in [69].

The VAI feature can be useful as a secondary risk factor; likely due to visceral fat being a consequence of OSA adversely influencing the systemic inflammation of the body, as observed by [70].

TyG was used as a predictor in this work, and the findings parallel the results of [71] where TyG had a noticeable independent correlation with OSA in both non-obese and non-diabetic patients.

Recent studies reveal the capability of sleep architecture, in terms of sleep stages and sleep duration, in producing effective technology enabled screening of sleep disorders. Sleep architecture is estimated by leveraging wearable sensors or smartwatches with machine learning methods and its effect on OSA screening is observed in [72,73]. Specifically, stage 1 and stage 3 sleep exhibited anomalous behavior in the case of OSA patients, as stated in [74,75,76]. Interestingly, the findings of our presented work does not reveal strong predictive powers when using the features of sleep stages (stage 1, stage 2, stage 3 and REM) as well as sleep duration metrics. This could be because OSA does not always reflect the same changes across all stages of sleep for all individuals, due to variations in pathophysiological factors such as airway collapsibility, muscle responsiveness, arousal thresholds, and stable ventilation. These points arise as substantial inconsistencies when conducting sleep experiments on populations with different demographical composition in terms of age, gender or ethnicity, as noted in [77,78]. This brings to light the need of extended monitoring to accurately confirm the severity of OSA in patients using sleep staging approaches as well.

The demographics in the dataset used in this work did not have many extreme cases of OSA, and the severities seem to be fairly imbalanced, in favor of mild and moderate cases. Despite the relatively older ages of the population (average age 58.02±8.04), OSA outcomes and associated medical conditions were not severe. A long-term study focusing on the same population as they age to analyze the OSA predictors and symptoms can likely reveal useful insights about the impact of lifestyle, and the potential consequences of other physiological and physical features. The OSA patient distribution was skewed towards men in this dataset. It could be due to the fact that women are generally less susceptible an OSA. As mentioned in [79], female hormones increases upper airway dilator muscle tone, and reduces the risk of pharangeal collapse (upper airway collapse), a major issue among OSA patients.

The presented work builds upon the findings reported previously in [21,22,60,61], which prove the feasibility of utilizing clinical information to screen for OSA patients and prioritize them for further sleep studies. Our models were able to predict clinical cases of OSA with reasonable accuracy, sensitivity and specificity, and is competitive with the recent electronic health record based prediction studies, as shown in Table 5. Consistent limitations in previous works include relatively fewer clinical parameters, high false positive rate, and demographic constraints. We observe that our proposed SVM model achieved the highest sensitivity among the existing works, with a specificity trade-off, in order to achieve a greater screening efficiency.

We further provide evidence that routinely clinical information can be effective in classification of OSA in a population health monitoring context. From the oximetry features, it can be said that desaturation severity, which consider the duration of apnea and hypopneas and the severity of breathing cessations may be more strongly related with daytime sleepiness and other symptoms than AHI or ODI [65,80]. Results suggest that oximetry data estimated using wearables, can be leveraged in conjunction with patient EHR to improve the detection rate, decrease false positives, and identify patients with risk of OSA. To enable continuous monitoring, another method would be to integrate personal health devices such as glucometers, in addition to the wearable data, as varying levels of glucose can indicate issues with metabolic issues concurrent with OSA complications. By considering all facets of an individual’s health, from associated comorbidities, to treatment and risk factors, machine learning models can reasonably indicate effective. By prioritizing patients based on symptom severity, physicians can verify which cases are urgent, and which cases are false alarms. The incorporation of specialist feedback can enable a continuous active learning process to continuously train and retrain the model for better predictability.

The limitations of these works are as follows. Low specificity when model is trained with EHR data, similar to previous works in this domain, as indicated by Table 5. Majority of the patients participating in the Wisconsin Sleep study have reported some symptoms of OSA. This leads to the prevalence being higher in this dataset than the general public, and there likely may be only minimal differences between the non-OSA and OSA populations. Furthermore, most cases in the mild severity category, where they may not be necessarily chronic, but perhaps intermittent and only exacerbated by underlying comorbidities. The Wisconsin Sleep Study was conducted over a span of 10 years with a single patient having up to five different entries, and as noted previously [39] increasing age is typically correlated with higher prevalence as well. The dataset used is saturated with the Caucasian demographic, which could hinder its applicability to other races.

## 5. Conclusions

Routinely available clinical information such as patient questionnaires responses and anthropometry can be used to develop screening obstructive sleep apnea (OSA) classification models. However, its relative effectiveness in comparison with models trained with physiological oximetry has not been established till this work. The purpose of this study was to incorporate additional clinical parameters such as laboratory blood tests, clinical surrogate markers and history of comorbidities for training machine learning models and empirically validate its performance against models trained on oximetry measures acquired from the same population. This study proposes a SVM for classifying OSA patients at the cut-off of apnea-hypopnea index ≥5 and achieved accuracy: 68.06%, sensitivity: 88.76%, specificity: 40.74%, F1-score: 75.96%, PPV: 66.36% and NPV: 73.33%, which is competitive with existing research. The findings of this study demonstrate the potential of screening models for the early detection of individuals with high pretest OSA possibility using routinely collected clinical parameters. To address the limitations of this work, a large-scale prospective study is likely needed to assess the performance of the proposed screening model on the general population.

## Figures and Tables

**Table 1:** Classification performance measures across ensemble and traditional models for 8 EHR features.

Model	*Acc%*	*Sen%*	*Sp%*	*F1-Score%*	*PPV%*	*NPV%*	*AUC%*
XGB	68.05	79.20	53.33	73.82	69.11	66.05	66.30
LGBM	67.41	74.15	58.52	72.13	70.21	63.20	66.33
CB	67.41	83.14	46.65	74.37	67.27	67.74	64.09
RF	68.05	77.52	55.55	73.40	69.69	65.22	66.54
kNN	67.09	77.00	54.00	72.67	68.84	64.03	65.55
LR	67.73	80.89	50.37	74.00	68.24	66.66	65.63
SVM	68.06	88.76	40.74	75.96	66.38	73.33	64.75

**Table 2:** Classification performance measures across ensemble and traditional models for 19 EHR features.

Model	*Acc%*	*Sen%*	*Sp%*	*F1-Score%*	*PPV%*	*NPV%*	*AUC%*
XGB	69.64	78.65	57.77	74.66	71.65	67.24	64.66
LGBM	68.37	73.60	61.48	72.57	71.58	63.84	67.53
CB	69.00	77.52	57.77	74.00	70.76	66.60	67.65
RF	65.81	73.03	56.30	70.84	68.78	61.30	64.66
kNN	63.25	69.10	55.55	68.14	67.21	57.69	62.32
LR	67.41	74.15	58.51	72.13	70.21	63.20	66.33
SVM	65.17	77.53	49.63	71.54	66.90	62.04	63.30

**Table 3:** Classification performance measures across ensemble and traditional models for 2 PSG features.

Model	*Acc%*	*Sen%*	*Sp%*	*F1-Score%*	*PPV%*	*NPV%*	*AUC%*
XGB	82.74	88.00	76.15	85.06	82.35	83.33	82.05
LGBM	83.04	87.42	77.48	85.20	83.08	83.00	82.97
CB	83.63	89.00	76.82	85.85	83.00	84.67	83.00
RF	83.63	87.43	78.80	85.64	84.00	83.20	83.12
kNN	82.74	88.48	75.50	85.13	82.03	83.82	82.00
LR	81.87	82.77	80.79	81.76	84.49	78.71	81.17
SVM	83.04	86.91	78.15	85.13	83.42	82.51	82.52

**Table 4:** Classification performance measures across ensemble and traditional models for 6 PSG features.

Model	*Acc%*	*Sen%*	*Sp%*	*F1-Score%*	*PPV%*	*NPV%*	*AUC%*
XGB	83.92	89.53	76.82	86.14	83.00	85.30	83.17
LGBM	83.33	88.50	76.82	85.56	82.84	84.05	82.65
CB	84.21	89.53	77.50	86.36	83.41	85.40	83.50
RF	84.50	89.53	78.14	86.60	83.82	85.50	86.58
kNN	83.33	88.00	77.48	85.50	83.17	83.57	82.72
LR	83.62	86.91	79.47	85.56	84.26	82.75	83.19
SVM	83.33	86.91	78.80	85.34	83.83	82.63	85.34

**Table 5:** A comparison of recent works developed for EHR-based screening of OSA through machine learning.

Source	Dataset	Features	Approach	*Sen%*	*Sp%*
This work	WSC ($n_{p}=940$)	waist-to-height ratio, waist circumference,	SVM	88.76	40.74
		neck circumference, BMI, EDS, LAP,			
		daily snoring frequency and snoring volume			
[21]	Private ($n_{p}=1922$)	age, hypertension, BMI and sex	SLIM	64.20	77.00
[22]	Private ($n_{p}=6875$)	waist circumference and age	SVM	74.14	74.71
[60]	Private ($n_{p}=279$)	waist circumference, frequency of falling asleep,	SVM	80.33	86.96
		subnasale to stomion length, hypertension,			
		snoring volume, and fatigue severity score			
[61]	Private ($n_{p}=313$)	BMI, ESS, and number of apneas	SVM	44.7	-

## Data Availability

The data presented in this study are openly available in National Sleep Research Resource at https://doi.org/10.25822/js0k-yh52, accessed on 16 September 2021.

## Abbreviations

The following abbreviations are used in this manuscript:

AHI	Apnea Hypopnea Index
ACC	Accuracy
AUC	Area Under Curve
BMI	Body-Mass Index
CB	Catboost Algorithm
CMA-ES	Covariance Matrix Adaptation Evolution Strategy
EDS	Excessive Daytime Sleepiness
ESS	Epworth Sleepiness Scale
EHR	Electronic Health Records
LAP	Lipid Accumulation Product
kNN	K-Nearest Neighbours
LGBM	Light Gradient Boosting
LR	Logistic Regression
ML	Machine Learning
MSLT	Multiple Sleep Latency Test
MWT	Maintenance of Wakefulness Test
NPV	Negative Predictive Value
OSA	Obstructive Sleep Apnea
PPV	Positive Predictive Value
PSG	Polysomnography
RF	Random Forest
SEN	Sensitivity
SLIM	Supersparse Linear Integer Model
SP	Specificity
SVM	Support Vector Machines
TPE	Tree-structured Parzen Estimator
WSC	Wisconsin Sleep Cohort
VAI	Visceral Adiposity Index
XGB	Extreme Gradient Boosting

## Appendix A

The complete code to reproduce this work and further details regarding the results of the statistical tests, participant IDs for training-validation and hold-out testing set split, and additional model pipeline configurations is available at https://github.com/jayrmh/EHRWSC, accessed on 8 October 2021.

**Table A1:** Demographic characteristics of cohort expressed as mean ± standard deviation.

Demographics	Overall	OSA (≥5)	No OSA (≤5)
	$n_{r}=1479$	$n_{r}=853$	$n_{r}=626$
AHI (/h)	12.26 ± 15.21	19.7 ± 16.33	2.03 ± 1.41
Age (y/o)	58.20 ± 8.04	59.483 ± 7.78	56.67 ± 8.12
Sex (%Male)	787 (53.2)	494 (57.81)	293 (46.08)
Race (%Caucasian)	1430 (96.68)	825 (96.71)	605 (96.65)
Alcohol (%Yes)	1080 (73.00)	619 (72.56)	461 (73.64)
Smoking (%Yes)	740 (50.00)	427 (50.00)	313 (50.00)

**Table A2:** Anthropometric characteristics of cohort expressed as mean ± standard deviation.

Anthropometric	Overall	OSA (≥5)	No OSA (≤5)
	$n_{r}=1479$	$n_{r}=853$	$n_{r}=626$
Height (cm)	169.04 ± 9.24	169 ± 9.19	168.70 ± 9.30
Weight (kg)	90.05 ± 20.50	95.27 ± 20.27	82.94 ±18.58
BMI (kg/m${}^{2}$)	31.54 ± 7.05	33.33 ± 7.29	29.09 ± 5.91
Neck Circumference (cm)	38.58 ± 4.04	39.53 ± 3.83	37.30 ± 3.966
Waist Circumference (cm)	99.89 ± 16.06	104.56 ± 15.25	93.55 ± 14.93

**Table A3:** Blood test profile characteristics of cohort expressed as mean ± standard deviation.

Blood Tests	Overall	OSA (≥5)	No OSA (≤5)
	$n_{r}=1479$	$n_{r}=853$	$n_{r}=626$
Height (cm)	169.04 ± 9.24	169 ± 9.19	168.70 ± 9.30
Weight (kg)	90.05 ± 20.50	95.27 ± 20.27	82.94 ±18.58
BMI (kg/m${}^{2}$)	31.54 ± 7.05	33.33 ± 7.29	29.09 ± 5.91
Neck Circumference (cm)	38.58 ± 4.04	39.53 ± 3.83	37.30 ± 3.966
Waist Circumference (cm)	99.89 ± 16.06	104.56 ± 15.25	93.55 ± 14.93

**Table A4:** Clinical surrogate marker characteristics of cohort expressed as mean ± standard deviation.

Clinical Surrogate Markers	Overall	OSA (≥5)	No OSA (≤5)
	$n_{r}=1479$	$n_{r}=853$	$n_{r}=626$
TyG	8.73 ± 0.60	8.82 ± 0.06	8.60 ± 0.58
LAP	340.12 ± 258.60	392.36 ± 268.20	268.92 ± 226.462
VAI	3.83 ± 3.07	4.21 ± 3.32	3.31 ± 2.66
Whrt	0.59 ± 0.09	0.61 ± 0.09	0.55 ± 0.08

**Table A5:** General health characteristics of cohort expressed as mean ± standard deviation.

General Health	Overall	OSA (≥5)	No OSA (≤5)
	$n_{r}=1479$	$n_{r}=853$	$n_{r}=626$
Zung Depression Scale	39.73 ± 8.13	40.07 ± 8.02	39.27 ±8.25
Horne Ostberg Score	62.40 ± 9.56	62.48 ± 9.84	62.25 ± 9.18
Epworth Sleepiness Scale	8.84 ± 4.17	9.22 ± 4.20	8.31 ± 4.08
State Anxiety Score	27.20 ± 6.91	27.11 ± 6.96	27.32 ±6.84
Trait Anxiety Score	31.67 ± 8.23	31.58 ± 8.15	31.76 ±8.33

**Table A6:** Comorbidities characteristics of cohort expressed as mean ± standard deviation.

Comorbidities	Overall	OSA (≥5)	No OSA (≤5)
	$n_{r}=1479$	$n_{r}=853$	$n_{r}=626$
Heart Attack (%yes)	61 (4.12)	50 (6.00)	11 (1.75)
Hypertension (%yes)	531 (36.00)	357 (41.80)	174 (27.79)
Arrhythmia (%yes)	203 (13.72)	126 (14.77)	77 (12.30)
Angina (%yes)	45 (3.40)	34 (4.00)	11 (1.75)
Coronary (%yes)	106 (7.16)	76 (8.90)	30 (4.79)
Atherosclerosis (%yes)	28 (1.90)	14 (1.64)	14 (2.23)
Congestive Heart Failure (%yes)	14 (0.09)	13 (0.16)	1 (1.52)
Asthma (%yes)	263 (17.70)	162 (18.99)	101 (16.13)
Emphysema (%yes)	24 (1.62)	13 (1.52)	11 (1.75)
Diabetes (%yes)	166 (11.22)	119 (13.95)	47 (7.50)
Stroke (%yes)	28 (1.90)	19 (2.22)	9 (1.43)
Thyroid (%yes)	195 (13.18)	112 (13.13)	83 (13.25)
Arthritis (%yes)	460 (31.10)	302 (35.40)	158 (25.23)
Sleep Apnea (%yes)	187 (12.64)	123 (14.42)	64 (10.22)

**Table A7:** Self-reported sleep characteristics of cohort expressed as mean ± standard deviation.

Sleep History	Overall	OSA (≥5)	No OSA (≤5)
	$n_{r}=1479$	$n_{r}=853$	$n_{r}=626$
Excessive Daytime Sleepiness	314 (21.23)	195 (22.86)	119 (19.00)
Sleep Latency (min)	14.78 ± 12.96	14.56 ± 11.51	15.13 ± 14.71
Trouble Falling Back to Sleep (%sometimes)	533 (36.03)	312 (36.57)	221 (35.30)
Feeling Not Rested (%rarely)	488 (33.00)	273 (32.00)	215 (34.34)
Waking Up Too Early (%rarely)	527 (35.63)	309 (36.22)	218 (24.82)
Waking Up Repeatedly (%rarely)	417 (28.19)	240 (28.13)	177 (28.27)
Difficulty Falling Asleep (%rarely)	612 (41.37)	358 (41.96)	254 (40.57)
Difficulty Waking Up (%rarely)	568 (38.40)	329 (38.56)	239 (38.17)
Frequency of Nightmares (%rarely)	666 (45.00)	393 (46.07)	273(43.61)
Frequency of Snoring (%every night)	393 (26.67)	300 (35.18)	93 (14.85)
Snoring Volume (%talkingvolume)	426 (28.80)	238 (30.03)	188 (28.00)
Sleep Satisfaction (%mostly)	1019 (68.89)	590 (69.16)	429 (68.53)

**Table A8:** PSG-derived oximetry characteristics of cohort expressed as mean ± standard deviation.

Oximetry	Overall	OSA (≥5)	No OSA (≤5)
	$n_{r}=1479$	$n_{r}=853$	$n_{r}=626$
Sleep Efficiency (%)	80.64 ± 10.16	79.67 ± 10.33	81.96±9.78
Sleep Latency (min)	12.63 ± 14.77	12.15 ± 14.91	13.30 ± 14.56
Average Oxygen Saturation (%)	95.32 ± 1.56	94.88 ± 1.58	95.90 ± 1.33
Minimum Oxygen Saturation (%)	85.00 ± 7.47	82.14 ± 7.72	88.89 ± 4.93
Average Oxygen Desaturation	4.54 ± 1.23	5.06 ± 1.35	3.83 ± 0.44
of Apnea-Hypopnea Event (%)			
Average Duration (s)	35.32 ± 8.58	33.85 ± 7.37	37.32 ± 9.65
of Apnea-Hypopnea Event			
Total Sleep Duration (min)	368.32 ± 57.45	364.12 ± 58.00	374.08 ± 56.22
REM Sleep Duration (min)	61.70 ± 25.92	58.35 ± 25.12	66.27 ± 26.30
REM Sleep Percentage (%)	16.51 ± 5.91	15.79 ±5.76	17.50 ±5.98
REM Latency (min)	123.40 ± 73.82	127.51 ± 76.06	117.96 ± 70.33
NREM Sleep Duration (min)	306.62 ± 47.65	305.75 ± 48.20	307.80 ± 46.90
Stage I and II Sleep Percentage (%)	76.21 ± 9.49	77.23 ± 9.31	74.82 ± 9.56
Stage III and IV Sleep Percentage (%)	7.26 ± 7.87	6.97±7.49	7.66 ±8.35
Wake After Sleep Onset (min)	68.89 ± 40.47	72.931 ± 40.93	63.38 ± 39.21
Sleep Duration Percentage	1.92 ± 8.16	2.94 ± 9.88	0.53 ± 4.56
with Oxygen Saturation below 90% (%)			

**Table A9:** Optimal hyperparameters for all ML models attained through tuning for the 8 feature EHR experiment.

Model	Hyperparameters
XGB	booster: dart
	lambda: $8.44\times 10^{-5}$
	alpha: $1.36\times 10^{-8}$
	max_depth: 4
	eta: 0.604
	gamma: 0.630
	grow_policy: depthwise
	sample_type: weighted
	normalize_type: forest
	rate_drop: 0.758
	skip_drop: $5.32\times 10^{-7}$
LGBM	booster: gbtree
	lambda: $4.18\times 10^{-8}$
	alpha: 0.166
	max_depth: 2
	*eta:* 0.005
	gamma: 0.007
	grow_policy: lossguide
CB	objective: logloss
	colsample_bylevel: 0.055
	depth: 9
	boosting_type: ordered
	bootstrap_type: MVS
RF	n_estimators: 610
	max_depth: 35
	min_samples_leaf: 55
	min_samples_split: 56
kNN	leaf_size: 70
	n_neighbors: 37
LR	C: 0.007
SVM	kernel: rbf
	gamma: 0.24
	C: 0.148

**Table A10:** Optimal hyperparameters for all ML models attained through tuning for 6 feature PSG experiment.

Model	Hyperparameters
XGB	booster: dart
	lambda: 0.0006
	alpha: 0.0003
	max_depth: 4
	eta: 0.009
	gamma: $3.838\times 10^{-5}$
	grow_policy: depthwise
	sample_type: weighted
	normalize_type: tree
	rate_drop: $1.2\times 10^{-8}$
	skip_drop: 0.0005
LGBM	booster: gbtree
	lambda: $4.23\times 10^{-6}$
	alpha: $3.76\times 10^{-7}$
	max_depth: 2
	eta: $1.14\times 10^{-8}$
	gamma: 0.914
	grow_policy: depthwise
CB	objective: crossentropy
	colsample_bylevel: 0.099
	depth: 4
	boosting_type: ordered
	bootstrap_type: Bernoulli
RF	n_estimators: 350
	max_depth: 79
	min_samples_leaf: 7
	min_samples_split: 10
kNN	leaf_size: 60
	n_neighbors: 63
LR	C: 2010.58
SVM	kernel: linear
	gamma: 5.68
	C: 1.657
